# Factors associated with sleep disorders in elderly patients with Parkinson’s disease and cognitive impairment and the nomogram model development

**DOI:** 10.3389/fnagi.2025.1670915

**Published:** 2026-01-05

**Authors:** Yimei Zhang, Liyan Sun, Haitao Chi

**Affiliations:** Department of Sleep Monitoring Room, Dalian University Affiliated Xinhua Hospital, Dalian, Liaoning Province, China

**Keywords:** Parkinson’s disease, cognitive impairment, sleep disorders, nomogram, risk prediction model, anxiety, depression, chronic pain

## Abstract

**Background:**

Sleep disorders are a common complication in elderly patients with Parkinson’s disease and cognitive impairment. This retrospective cohort study investigates the factors associated with sleep disorders in elderly patients with Parkinson’s disease and cognitive impairment and proposes a framework for a future comprehensive relaxation training intervention based on the identified factors, to inform subsequent clinical studies.

**Methods:**

A retrospective study was conducted on 108 elderly patients with Parkinson’s disease and cognitive impairment who visited the outpatient department of our hospital from January 2021 to December 2024. All patient data were obtained from the electronic medical record system. Patients were divided into a sleep disorder group (*n* = 40) and a non-sleep disorder group (*n* = 68) based on the presence or absence of sleep disorders. General information differences between the two groups were collected and compared. Collinearity analysis was performed on indicators with significant differences between the two groups. Logistic regression analysis was used to identify the primary factors associated with sleep disorders in elderly patients with Parkinson’s disease and cognitive impairment. A line chart was established using R software for validation. Finally, a framework for a comprehensive relaxation training intervention was proposed as a potential future clinical application based on the model’s findings.

**Results:**

There were statistically significant differences between the sleep disorder group and the non-sleep disorder group in terms of Hoehn-Yahr staging, equivalent dose of levodopa, Hamilton Anxiety Scale (HAMA), Hamilton Depression Scale (HAMD), and chronic pain (*p* < 0.05). No collinearity was observed among the indicators. Multivariate logistic regression analysis revealed that Hoehn-Yahr staging, equivalent dose of levodopa, HAMA, HAMD, and chronic pain were all risk factors for sleep disorders in elderly Parkinson’s disease patients with cognitive impairment (OR = 6.327, 2.698, 3.203, 1.041, 1.217, *p* < 0.05). Based on the results of the logistic regression analysis, a risk prediction nomogram model for sleep disorders in elderly patients with Parkinson’s disease and cognitive impairment was constructed. The receiver operating characteristic (ROC) curve showed an area under the curve (AUC) value of 0.963 (95% CI, 0.931–0.955). The calibration curve indicated that the model’s predictive results were well aligned with the actual occurrence of sleep disorders in elderly patients with Parkinson’s disease and cognitive impairment, with a Brier Score of 0.051 and a model fit *p*-value of 0.925. The statistic was 2.688. The clinical decision curve was generally higher than the two extreme curves, indicating that the factors included in the plot diagram have a high net benefit in predicting sleep disorders in elderly patients with Parkinson’s disease cognitive impairment.

**Conclusion:**

There are numerous factors associated with sleep disorders in elderly patients with Parkinson’s disease and cognitive impairment, primarily including Hoehn-Yahr staging, equivalent dose of levodopa, HAMA, HAMD, and chronic pain. The risk prediction nomogram model constructed based on these factors has certain predictive value for the occurrence of sleep disorders, can assist in the early screening of high-risk populations in clinical practice, and provides a basis for developing corresponding relaxation training interventions to reduce the occurrence of sleep disorders.

## 1 Introduction

Parkinson’ s disease (PD) is the second most prevalent neurodegenerative disorder globally, imposing a significant burden on the aging population (Ojo et al., 2024; Hauser et al., 2023). While motor symptoms remain the diagnostic hallmark, non-motor symptoms—particularly cognitive impairment and sleep disorders—are increasingly recognized as critical determinants of disease burden and quality of life (Mantovani et al., 2023). Sleep disorders may indicate cognitive impairment and can also manifest as a concomitant symptom of cognitive impairment. Additionally, impaired sleep quality can further exacerbate cognitive impairment (Maggi et al., 2021; Marafioti et al., 2023).

Sleep disorders in PD are highly heterogeneous, encompassing insomnia, excessive daytime sleepiness, and REM sleep behavior disorder, among others (Lajoie et al., 2021; Taximaimaiti et al., 2021). Their underlying mechanisms are multifactorial, involving neurodegenerative changes across monoaminergic and cholinergic systems, neuropsychiatric comorbidities, medication effects, and chronic pain (Iranzo et al., 2024; Thangaleela et al., 2023). In addition, negative emotions such as anxiety and depression can directly impact the sleep quality of Parkinson’s disease patients, while sleep disorders can exacerbate cognitive impairments and frailty in these patients, thereby intensifying negative emotions (Thangaleela et al., 2023; Qiu et al., 2022). Although several studies have attempted to identify risk factors for sleep disorders in general PD cohorts, few have specifically focused on the high-risk subgroup of elderly patients with concomitant cognitive impairment (Prajjwal et al., 2023). Consequently, a clear consensus on the independent risk factors in this vulnerable population is still lacking.

This knowledge gap directly impedes the development of targeted non-pharmacological interventions. Comprehensive relaxation training—a multimodal approach integrating breathing, muscular, and mindfulness techniques—represents a promising strategy to address several modifiable risk factors, such as anxiety, depression, and pain (Hampson et al., 2020). However, the design of such interventions has seldom been informed by robust, model-derived risk profiles, limiting their clinical applicability and efficacy.

Therefore, this study was designed with the following objectives: First, we aimed to identify independent risk factors for sleep disorders in elderly PD patients with cognitive impairment using a rigorous statistical workflow, including univariate screening with Bonferroni correction followed by multivariate logistic regression. Second, based on the identified factors, we constructed a clinically practical nomogram model to facilitate individualized risk prediction. Finally, we systematically formulated a comprehensive relaxation training intervention specifically tailored to address the modifiable risk factors embedded in the model, thereby providing an evidence-based, mechanism-informed framework for managing sleep disturbances in this complex patient population.

## 2 Materials and methods

### 2.1 Ethical statement

This study was approved by the Institutional Review Board and Ethics Committee of Dalian University Affiliated Xinhua Hospital (No. 2025-079-01). Given that this study is a retrospective study and only de-identified patient data was used, informed consent was not required as there was no risk or adverse effect on patient care. This exemption complies with regulations and ethical guidelines related to retrospective studies.

### 2.2 Study design

This is a single-center retrospective cohort study. This retrospective analysis included 108 elderly patients with Parkinson’s disease cognitive impairment who visited the neurology outpatient department of our hospital from January 2021 to December 2024. All patient data were obtained from the electronic medical record system and were divided into a sleep disorder group (*n* = 40) and a non-sleep disorder group (*n* = 68) based on the presence or absence of sleep disorders.

### 2.3 Inclusion criteria

Inclusion criteria: (1) All patients met the 2015 Parkinson’s disease Diagnostic Criteria issued by the International Movement Disorder Society (MDS) (Postuma et al., 2015); (2) age ≥ 65 years; (3) Montreal Cognitive Assessment (MoCA) (Nasreddine et al., 2005) score <26 points; (4) good compliance, able to complete the scale assessment; (5) complete data, and accessible for review. Exclusion criteria: (1) Parkinson’s syndrome caused by vascular, intracranial infection, trauma, drugs, or toxins, as well as Parkinson’s superimposed syndrome; (2) patients with severe organ dysfunction of the heart, liver, or lungs; (3) patients with malignant tumors; (4) patients with pre-existing sleep disorders; (5) patients with painful diseases, drug, or alcohol addiction.

### 2.4 General data collection

General demographic data on patients were retrospectively collected through the electronic medical record system, primarily including gender, age, educational attainment, body mass index (BMI), smoking history (defined as daily smoking volume > 1 cigarette, with a continuous duration > 1 year or cessation period < 1 year) (yes/no), alcohol consumption history (defined as daily alcohol intake > 1 drink unit, duration > 1 year, or abstinence < 1 year, where 1 drink unit = 45 mL of spirits/360 mL of beer/120 mL of wine) (yes/no), hypertension (yes/no), diabetes (yes/no), disease duration, equivalent dose of levodopa tablets (equivalent dose of levodopa calculated as entacapone = levodopa × 0.33, 1 mg rasagiline = 1 mg, pramipexole = 10 mg, selegiline = 100 mg, piribedil = 100 mg, levodopa standard tablets = 133 mg levodopa controlled-release tablets). The presence or absence of chronic pain was retrospectively determined by the research team based on the Ford classification system. Patient records were reviewed for documented symptoms consistent with central pain, skeletal muscle pain, radicular pain, pain caused by akathisia/restlessness, or dystonic pain. The presence of any of these symptoms led to classification as having chronic pain (Buhmann et al., 2020).

### 2.5 Hoehn-Yahr staging

The Hoehn-Yahr staging system classifies patients into five stages based on disease severity: Stage 1 involves unilateral involvement; Stage 2 involves bilateral involvement but no postural balance disorders; Stage 3 involves the onset of postural balance disorders, often leading to frequent falls; Stage 4 requires assistance with daily activities and difficulty walking independently; Stage 5 involves nearly constant reliance on a wheelchair or inability to rise from a seated position throughout the day. Stages 1–2 are classified as mild, while stages 3–5 are classified as moderate to severe (Shen et al., 2022).

### 2.6 Psychological status assessment

As part of the routine clinical assessment in our Parkinson’s disease outpatient clinic, patients’ psychological status was evaluated using standardized scales. The Hamilton Anxiety Scale (HAMA) (He et al., 2024) is used to assess patients, consisting of 14 items, each scored out of 4 points, with a total score of 56 points. The score is positively correlated with the severity of the patient’s anxiety. The Hamilton Depression Rating Scale (HAMD) (Li et al., 2025) is used for assessment, comprising 17 items, each scored out of 4 points, with a total score of 68 points. The score is directly proportional to the severity of the patient’s depressive symptoms.

### 2.7 Cognitive function assessment

The Chinese version of the Montreal Cognitive Assessment (MoCA, Beijing Revision) (Niu et al., 2025) was used to assess patients. An additional point was added to the total score for patients with 12 or fewer years of education. The use of the MoCA in this non-commercial academic study, where all assessments were provided free of charge, is compliant with the official user policy and did not require separate written authorization. The scale includes visuospatial and executive function (5 points), naming (3 points), memory (not scored), attention (6 points), language (3 points), abstraction (2 points), delayed recall (5 points), and orientation (6 points), with a total score of 30 points. Higher scores indicate better cognitive function, and a score <26 suggests possible cognitive impairment.

### 2.8 Sleep disorder assessment

Patients were assessed using the Pittsburgh Sleep Quality Index (PSQI), which includes 19 self-rated items and 5 other-rated items, namely sleep quality, sleep onset latency, sleep duration, sleep efficiency, sleep disturbances, hypnotic medication use, and daytime functioning. Each component is scored on a 0–3 scale, and the total score is the sum of all components. The total score ranges from 0 to 21 points, with higher scores indicating poorer sleep quality. A total PSQI score ≥8 was adopted as the cutoff to define poor sleep quality (Liu et al., 2021). In this study, patients with a PSQI total score ≥8 were assigned to the sleep disorder group, and those with a PSQI total score <8 were assigned to the non-sleep disorder group. Additionally, the presence of REM sleep behavior disorder (RBD) was evaluated. Screening was performed using the REM Sleep Behavior Disorder Screening Questionnaire (RBDSQ). Patients with a positive screening result (typically an RBDSQ score ≥5) subsequently underwent overnight polysomnography (PSG) for confirmatory diagnosis, in accordance with the International Classification of Sleep Disorders, Third Edition (ICSD-3) criteria.

### 2.9 Statistical methods

A *post hoc* power analysis was conducted using G*Power 3.1. With an alpha level of 0.05 and an effect size (*d*) of 0.6, the achieved power for the current sample (*n* = 108) was 0.85, indicating acceptable statistical power to detect medium-to-large effects. Data were analyzed using SPSS 25.0 statistical software. Categorical data were expressed as n, and compared using the Chi-square test. Continuous data were tested for normality using the Shapiro–Wilk test. Normally distributed data were presented as mean ± standard deviation (SD) and compared using the independent samples *t*-test; non-normally distributed data were expressed as median (interquartile range) and compared using the Mann–Whitney *U*-test. No missing data were present in the dataset; hence, no imputation was performed. Collinearity analysis was conducted for indicators showing significant differences between the two groups. To identify potential risk factors, univariate logistic regression analyses were first performed for all baseline variables. Variables with a *p*-value < 0.05 in the univariate analysis were then included in a multivariate logistic regression model. To account for multiple testing in the univariate analyses, Bonferroni correction was applied. The results of the multivariate analysis are presented as odds ratios (ORs) with 95% confidence intervals (CIs). A prediction model based on a scatter plot developed using the rms package was validated internally through 1,000 bootstrap resampling iterations. The area under the receiver operating characteristic (ROC) curve (AUC) was calculated to evaluate discriminative ability, calibration curves were plotted to assess predictive accuracy, and decision curves were applied to evaluate clinical utility. *p* < 0.05 was considered statistically significant.

### 2.10 Development of the comprehensive relaxation training intervention

Based on the identified risk factors for sleep disorders in this cohort—particularly anxiety/depression (elevated HAMA/HAMD), chronic pain, and advanced disease severity (higher Hoehn-Yahr stage)—a multimodal relaxation intervention was systematically designed to address these specific therapeutic targets. The program integrates four evidence-based techniques, each selected for their complementary mechanisms: diaphragmatic breathing exercises to regulate autonomic arousal and alleviate anxiety; progressive muscle relaxation to reduce musculoskeletal tension and pain perception; relaxation music therapy to promote emotional regulation and sleep-onset facilitation; and adapted yoga postures (e.g., Child’s Pose, butterfly pose) to address both physical rigidity and psychological stress. Each supervised session will last approximately 40 min, administered three times per week by trained rehabilitation therapists, with supplementary home-based practice encouraged using provided audio-visual materials. This combined approach aims to concurrently modulate physiological hyperarousal, improve pain coping, and enhance psychological well-being, thereby targeting the multifactorial nature of sleep disturbances in this population.

## 3 Results

### 3.1 Comparison of general characteristics between the sleep disorder group and the non-sleep disorder group

There were no statistically significant differences between the sleep disorder group and the non-sleep disorder group in terms of gender, age, educational level, BMI, medical history, disease duration, and MoCA scores (*p* > 0.05). However, there were significant differences in Hoehn-Yahr staging (11:29 vs. 48:20), equivalent doses of levodopa (452.33 ± 42.78 vs. 357.78 ± 41.96) mg/day, HAMA (28.75 ± 3.78 vs. 24.76 ± 4.54) points, HAMD (35.52 ± 5.30 vs. 31.84 ± 3.56) points, and chronic pain (14:26 vs. 5:63) showed statistically significant differences (*p* < 0.05), as shown in Table 1.

**Table 1:** Comparison of general information between the sleep disorder group and the non-sleep disorder group.

Index		Sleep disorder group (*n* = 40)	Non-sleep disorder group (*n* = 68)	χ^2^/*t/Z* value	*p-*value
General demographic data					
Sex (*n*)	Male	25	40	0.142	0.706
	Female	15	28		
Age ( $\bar{\chi }$ ± s, years)		67.50 ± 5.64	68.12 ± 5.78	0.543	0.588
Education (*n*)	Junior high school or below	22	39	0.057	0.812
	High school or above	18	29		
BMI ( $\bar{\chi }$ ± s, kg/m^2^)		21.74 ± 2.56	21.95 ± 2.48	0.420	0.675
Previous history					
Smoking history (*n*)		10	15	0.122	0.726
Drinking history (*n*)		6	9	0.066	0.798
High blood pressure (*n*)		8	13	0.013	0.911
Diabetes (*n*)		7	11	0.032	0.859
Clinical features					
Course of disease ( $\bar{\chi }$ ± s, years)		4.78 ± 1.25	4.62 ± 1.35	0.611	0.542
Stage of Hoehn-Yahr (*n*)	1 ~ 3 stage	11	48	18.865	<0.001
	4 ~ 5 stage	29	20		
HAMA ( $\bar{\chi }$ ± s, scores)		28.75 ± 3.78	24.76 ± 4.54	4.683	<0.001
HAMD ( $\bar{\chi }$ ± s, scores)		35.52 ± 5.30	31.84 ± 3.56	3.379	0.001
MoCA ( $\bar{\chi }$ ± s, scores)		20.44 ± 3.25	19.85 ± 3.34	0.886	0.378
Equivalent dose of levodopa tablets ( $\bar{\chi }$ ± s, mg/d)		452.33 ± 42.78	357.78 ± 41.96	11.227	<0.001
Chronic pain (*n*)	yes	14	5	13.278	<0.001
	no	26	63		

BMI, Body Mass Index; HAMA, Hamilton Anxiety Scale; HAMD, Hamilton Depression Scale; MoCA, Montreal Cognitive Assessment. Categorical data were expressed as *n*, and compared using the chi-square test. Continuous data were tested for normality using the Shapiro–Wilk test. Normally distributed data were presented as mean ± standard deviation (SD) and compared using the independent samples *t*-test.

### 3.2 Assignment table for study variables

Univariate logistic regression analyses were performed for all variables outlined in Table 2 to screen for potential factors associated with sleep disorders. The results (Table 3) indicated that Hoehn-Yahr stage, levodopa equivalent dose, HAMA score, HAMD score, and chronic pain were significantly associated with sleep disorders (all *p* < 0.05). In contrast, variables such as sex, age, education level, BMI, smoking history, drinking history, hypertension, diabetes, disease duration, and MoCA score showed no significant association (all *p* > 0.05).

**Table 2:** Assignment table for research variables.

Index	Type	Assignment method
The occurrence of sleep disorders in elderly patients with cognitive impairment caused by Parkinson’s disease	Binary	0 = not occurred, 1 = occurred
Sex	Binary	0 = Female, 1 = male
Age	Continuous	Measured value
Education	Binary	0 = Junior high school or below, 1 = high school or above
BMI	Continuous	Measured value
Smoking history	Binary	0 = No, 1 = yes
Drinking history	Binary	0 = No, 1 = yes
High blood pressure	Binary	0 = No, 1 = yes
Diabetes	Binary	0 = No, 1 = yes
Course of disease	Continuous	Measured value
Stage of Hoehn-Yahr	Binary	0 = 1 ~ 3 stage, 1 = 4 ~ 5 stage
Equivalent dose of levodopa tablets	Continuous	Measured value
HAMA	Continuous	Measured value
HAMD	Continuous	Measured value
MoCA	Continuous	Measured value
Chronic pain	Binary	Measured value

BMI, Body Mass Index; HAMA, Hamilton Anxiety Scale; HAMD, Hamilton Depression Scale; MoCA, Montreal Cognitive Assessment.

**Table 3:** Univariate logistic regression analysis of factors associated with sleep disorders.

Index	Β value	SE	Wald *χ*^2^	*p*-value	OR value	95%CI
Sex	−0.154	0.405	0.145	0.704	0.857	0.388 ~ 1.894
Age	0.021	0.037	0.322	0.570	1.021	0.949 ~ 1.099
Education	0.125	0.412	0.092	0.762	1.133	0.505 ~ 2.540
BMI	0.041	0.081	0.256	0.613	1.042	0.889 ~ 1.221
Smoking history	0.288	0.456	0.399	0.528	1.333	0.546 ~ 3.257
Drinking history	0.357	0.542	0.434	0.510	1.429	0.494 ~ 4.133
High blood pressure	0.185	0.478	0.150	0.699	1.203	0.472 ~ 3.069
Diabetes	0.288	0.508	0.321	0.571	1.333	0.493 ~ 3.606
Course of disease	0.061	0.101	0.365	0.546	1.063	0.873 ~ 1.294
Stage of Hoehn-Yahr	1.686	0.391	18.590	<0.001	5.396	2.507 ~ 11.615
Equivalent dose of levodopa tablets	0.022	0.004	32.786	<0.001	1.022	1.014 ~ 1.030
HAMA	0.226	0.049	21.253	<0.001	1.254	1.139 ~ 1.380
HAMD	0.140	0.047	8.861	0.002	1.150	1.049 ~ 1.261
MoCA	−0.056	0.061	0.845	0.358	0.946	0.839 ~ 1.066
Chronic pain	1.435	0.408	12.371	<0.001	4.200	1.014 ~ 1.030

Significance level set at *α* = 0.003 (Bonferroni correction). BMI, Body Mass Index; HAMA, Hamilton Anxiety Scale; HAMD, Hamilton Depression Scale; MoCA, Montreal Cognitive Assessment.

### 3.3 Results of multicollinearity analysis

Before constructing the multivariate model, multicollinearity analysis was assessed for the five significant variables identified in the univariate analysis revealed that there was no multicollinearity among Hoehn-Yahr staging, equivalent dose of levodopa, HAMA, HAMD, and chronic pain (VIF ≤ 10, tolerance ≥ 0.1). Therefore, all five variables were eligible for inclusion in the subsequent multivariate logistic regression model, as shown in Table 4.

**Table 4:** Results of collinearity analysis.

Index	VIF	Tolerance
Stage of Hoehn-Yahr	1.525	0.656
Equivalent dose of levodopa tablets	9.186	0.109
HAMA	6.373	0.157
HAMD	7.025	0.142
Chronic pain	1.462	0.684

HAMA, Hamilton Anxiety Scale; HAMD, Hamilton Depression Scale.

### 3.4 Multivariate logistic regression analysis of sleep disorders in elderly Parkinson’s disease patients with cognitive impairment

The five significant variables from the univariate analysis were incorporated into a multivariate logistic regression model. it was found that Hoehn-Yahr staging, equivalent dose of levodopa, HAMA, HAMD, and chronic pain were all risk factors for sleep disorders in elderly patients with Parkinson’s disease and cognitive impairment (OR = 6.327, 2.698, 3.203, 1.041, 1.217, *p* < 0.05), indicating that Hoehn-Yahr staging, equivalent dose of levodopa, HAMA, HAMD, and chronic pain are closely associated with sleep disorders in elderly patients with Parkinson’s disease and cognitive impairment, as shown in Table 5.

**Table 5:** Multivariate logistic regression analysis of independent risk factors for sleep disorders.

Index	Β value	SE	Wald *χ*^2^	*p*-value	OR value	95%CI
Stage of Hoehn-Yahr	1.845	0.443	17.345	0.001	6.327	2.656 ~ 15.076
Equivalent dose of levodopa tablets	0.992	0.399	6.180	0.013	2.698	1.234 ~ 5.898
HAMA	1.164	0.486	5.736	0.017	3.203	1.236 ~ 8.303
HAMD	0.041	0.016	6.397	0.011	1.041	1.009 ~ 1.075
Chronic pain	0.197	0.046	18.594	0.000	1.217	1.113 ~ 1.331

HAMA, Hamilton Anxiety Scale; HAMD, Hamilton Depression Scale.

### 3.5 Establishment and validation of the risk prediction regression model

**Figure 1:**
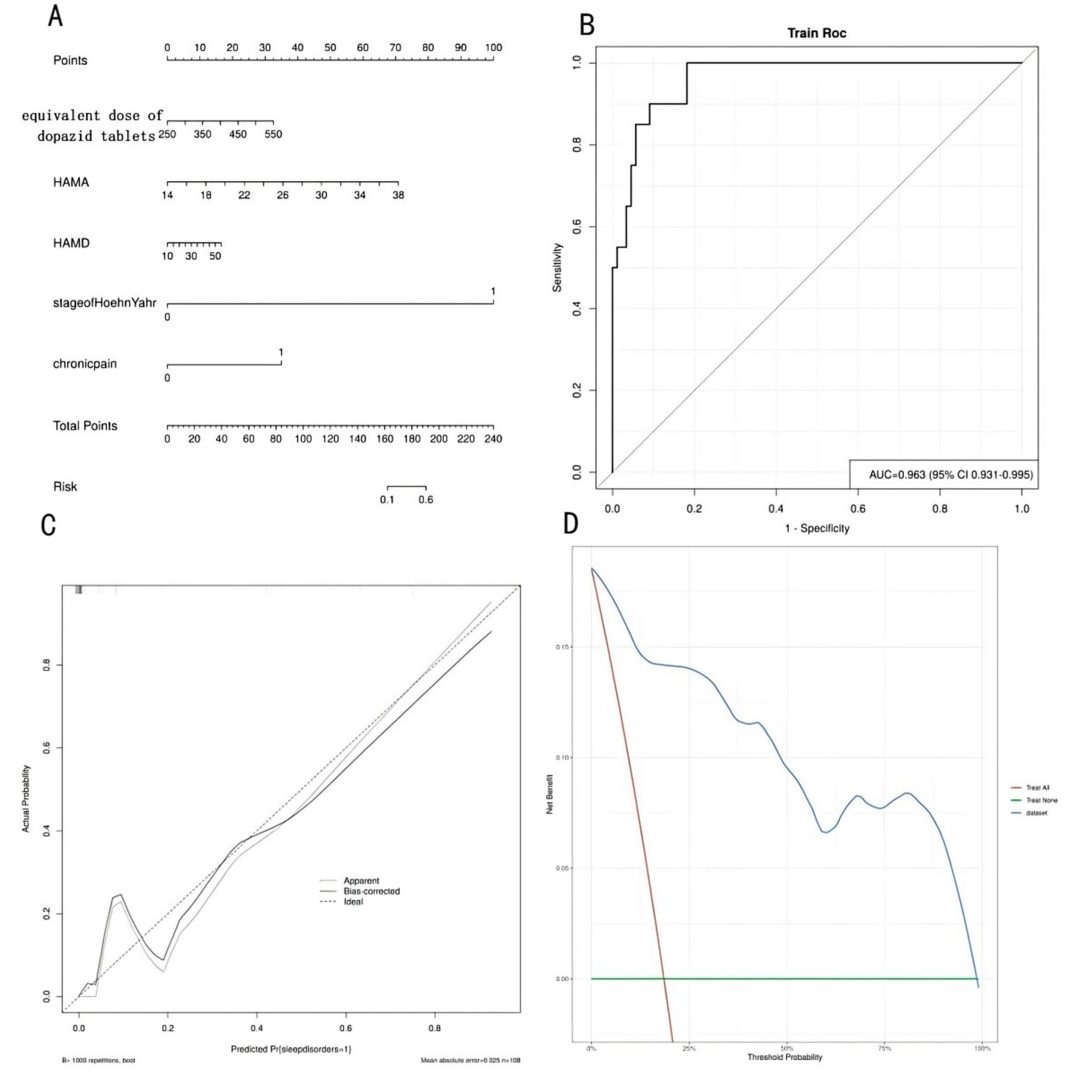
Establishment and validation of the risk prediction nomogram model. **(A)** The risk prediction nomogram model for sleep disorders in elderly patients with Parkinson’s disease and cognitive impairment. **(B)** The ROC curve of the risk prediction nomogram model. **(C)** The calibration curve of the risk prediction nomogram model. **(D)** The decision curve of the risk prediction nomogram model.

Based on the results of logistic regression analysis, a risk prediction regression model for sleep disorders in elderly patients with Parkinson’s disease and cognitive impairment was constructed. According to the patients’ Hoehn-Yahr staging (first row), equivalent dose of levodopa (second row), HAMA (third row), HAMD (fourth row), and chronic pain (fifth row), the corresponding values are located on the respective axes and projected vertically upward to the Points (score) axis to obtain individual scores; the three scores are then summed, and the corresponding position is located on the Total Points (total score) axis. Project vertically downward from the Total Points axis to the Risk axis to predict the probability of sleep disorders in elderly Parkinson’s disease patients with cognitive impairment, as shown in Figure 1A. By plotting the ROC curve, the AUC value is 0.963, with a 95% CI of 0.931 to 0.955, indicating that the nomogram model has good discriminative ability, as shown in Figure 1B. The model was validated using the Bootstrap method with 1,000 repeated samples. The Brier Score was 0.051, the model fit *p*-value was 0.925, and the statistic was 2.688, indicating that the column chart model exhibits good calibration, as shown in Figure 1C. The decision curve is higher than the two extreme curves, indicating that the net predictive gain of the relevant factors in the column chart is high, as shown in Figure 1D.

## 4 Discussion

Cognitive impairment is a common non-motor symptom in elderly patients with Parkinson’s disease. Elderly patients with Parkinson’s disease who also have cognitive impairment are prone to sleep disorders due to various factors such as the progression of the disease. Sleep disorders manifest in various forms, with common symptoms including insomnia, excessive daytime sleepiness, periodic limb movements, restless legs syndrome, and sleep-disordered breathing (Scanga et al., 2023). Although sleep disorders are relatively common in clinical practice, they are often overlooked, and there has been no unified standardized treatment protocol to date (Qian et al., 2025). Therefore, understanding the risk factors for sleep disorders in elderly Parkinson’s disease patients with cognitive impairment can facilitate targeted clinical interventions and improve patient outcomes. This study found that Hoehn-Yahr staging, equivalent doses of levodopa, HAMA, HAMD, and chronic pain all influence the occurrence of sleep disorders in elderly Parkinson’s disease patients with cognitive impairment. Additionally, risk prediction models based on these factors can assist clinicians in early screening for elderly Parkinson’s disease patients with cognitive impairment who are at higher risk of sleep disorders, and tailor relaxation training programs based on the results to improve patients’ sleep quality.

This study found that the Hoehn-Yahr staging, equivalent dose of levodopa, and chronic pain were higher in the sleep disorder group than in the non-sleep disorder group. Furthermore, Hoehn-Yahr staging, equivalent dose of levodopa, and chronic pain were all risk factors for the occurrence of sleep disorders in elderly Parkinson’s disease patients with cognitive impairment. The Hoehn-Yahr staging system assesses the severity of Parkinson’s disease. Patients with higher staging experience postural balance disorders, muscle rigidity, and bradykinesia, leading to difficulty turning over at night and positional transfer disorders, which cause frequent awakenings and disrupt sleep continuity, thereby resulting in sleep disorders (Happe et al., 2002). This finding aligns with previous studies indicating that advanced disease stages are strongly correlated with sleep fragmentation and reduced sleep efficiency (Kataoka et al., 2020). Carbidopa-levodopa is a combination of levodopa and carbidopa. Previous studies have shown (Antonini et al., 2021) that carbidopa-levodopa has a bidirectional regulatory effect on sleep: high doses can cause sleep disruption and prolonged sleep latency, while low doses can improve sleep. Therefore, a high equivalent dose of carbidopa-levodopa is associated with an increased risk of sleep disorders in patients. Our results are consistent with the literature, further confirming that the dosage of levodopa-carbidopa must be carefully optimized to minimize its adverse effects on sleep (Grétarsdóttir et al., 2021). Patients with chronic pain experience pain signals invading the sleep center, leading to central nervous system sensitization, with the brain in a state of heightened alertness, making it difficult to enter sleep or causing awakening due to pain during sleep (Li et al., 2024). Therefore, elderly Parkinson’s disease patients with cognitive impairment and chronic pain have a higher risk of developing sleep disorders. This is consistent with recent studies highlighting the role of nociceptive pathways in disrupting sleep architecture in PD (Lynch et al., 2024).

Clinical studies have shown (Lian et al., 2024; Li et al., 2022) that chronic inflammatory responses are present in the brains of Parkinson’s disease patients. This inflammation may exacerbate neuronal damage and impair the synthesis and release of neurotransmitters, leading to mood disorders and significantly increasing the incidence of anxiety and depression. This study found that the HAMA and HAMD scores were higher in the sleep disorder group than in the non-sleep disorder group, and that HAMA and HAMD scores were also risk factors for sleep disorders in elderly Parkinson’s disease patients with cognitive impairment. Further supporting this, recent evidence suggests that neuroinflammation may serve as a common pathophysiological link between affective symptoms and sleep disruption in PD (Yu et al., 2013). Patients’ own anxiety and depression can lead to abnormal secretion of neurotransmitters such as dopamine, norepinephrine, and serotonin, which are also involved in the occurrence and development of sleep disorders (Wu et al., 2024). Abnormal secretion of neurotransmitters increases the risk of sleep disorders in elderly Parkinson’s disease patients, further confirming the conclusions of this study.

The nomogram model developed in this study integrates these five key predictors (Hoehn-Yahr stage, levodopa equivalent dose, HAMA, HAMD, and chronic pain) into a clinically accessible tool. The model demonstrated excellent discriminative ability (AUC = 0.963) and calibration (Brier score = 0.051), indicating its potential utility in routine clinical practice. By providing individualized risk estimates, the nomogram can assist clinicians in identifying high-risk patients who may benefit from early non-pharmacological interventions, such as the comprehensive relaxation training proposed herein. This aligns with the growing emphasis on personalized medicine in chronic neurological disorders (Gotovac et al., 2014).

Based on the above results, the following comprehensive relaxation training strategies are proposed and will be implemented in future work: (1) During outpatient follow-up visits, guide patients to undergo breathing relaxation training, informing them that deep breathing can help relax the body and alleviate discomfort and pain caused by chest tube placement. Demonstrate proper diaphragmatic breathing and slow, deep breathing techniques to stabilize the patient’s emotions. (2) Progressive muscle relaxation training: Guide patients to consciously relax all muscles in the body, starting from the head and gradually moving down to the toes, while coordinating with deep breathing. This helps alleviate anxiety and depression caused by pain. (3) Relaxation music therapy: Select appropriate music based on the patient’s preferences, guide patients to listen to music before bedtime to aid sleep, adopt a comfortable lying position, close their eyes, relax the entire body, and promote emotional stability. (4) Yoga relaxation training: Instruct patients on proper yoga poses such as the Child’s Pose and Butterfly Pose and help them master breathing techniques. Use yoga poses to relax the mind and body and reduce psychological stress. After yoga training, instruct patients to meditate by closing their eyes, focusing their attention on their breathing, and feeling each part of their body gradually relax, thereby stabilizing their emotions and alleviating anxiety and depression.

However, this study has certain limitations. First, the sample size of 108 was formed through consecutive enrollment of eligible patients from our outpatient clinic between January 2021 and December 2024, constituting a convenience sample rather than one determined by *a priori* power calculation. Although *post hoc* analysis indicated acceptable statistical power (0.85) for detecting medium-to-large effects (*α* = 0.05, effect size d = 0.6), the generalizability of the findings may be constrained by the single-center, tertiary-hospital setting. Patients in such settings often present with more advanced disease stages and greater comorbidity burdens than those in community-based practices, potentially limiting the direct applicability of our results to broader populations. Second, the retrospective and cross-sectional design inherently restricts causal inference. The associations identified—such as those between higher HAMA/HAMD scores and sleep disorders—reflect temporal correlations rather than established causation. It remains unclear whether anxiety and depression contribute to sleep disturbances, whether poor sleep exacerbates affective symptoms, or whether both arise from shared neuropathological mechanisms in Parkinson’s disease. Similarly, the directional relationships among disease progression, medication use, chronic pain, and sleep disorders warrant further clarification through longitudinal studies. Additionally, the proposed comprehensive relaxation training intervention, though grounded in the identified risk profile, has not yet been empirically evaluated. Future studies should prioritize multi-center collaborations with larger, prospectively recruited cohorts to externally validate the nomogram, incorporate a wider range of potential influencing factors, and assess the short- and long-term efficacy of the tailored intervention in improving sleep outcomes. Despite these limitations, this study provides an integrative and interdisciplinary framework—bridging neurology, psychology, and rehabilitation—for understanding and addressing sleep disorders in elderly PD patients with cognitive impairment. The proposed relaxation strategies, targeting both physiological and psychological dimensions, offer a structured foundation for future intervention trials aimed at improving sleep quality and overall well-being in this vulnerable population.

## 5 Conclusion

In this retrospective cohort study, Hoehn-Yahr stage, levodopa equivalent dose, HAMA score, HAMD score, and chronic pain were identified as independent risk factors for sleep disorders in elderly patients with Parkinson’s disease and cognitive impairment. The risk prediction nomogram model constructed based on these factors demonstrates predictive value for the occurrence of sleep disorders. This model can assist clinicians in the early screening of high-risk individuals and, more importantly, serves as a foundational tool for developing personalized comprehensive relaxation training interventions. These tailored interventions, focusing on modifiable factors like anxiety, depression, and pain, offer a promising strategy to reduce the incidence of sleep disorders and improve the quality of life in this vulnerable population.

## Data Availability

The original contributions presented in the study are included in the article/supplementary material, further inquiries can be directed to the corresponding author.
